# Medullary Sarcoma of the Liver

**Published:** 1858-03

**Authors:** John P. Kluge

**Affiliations:** Physician to the Panama R. R. Company, and formerly Resident Physician at the Emigrant’s Refuge Hospital, New York


ART. IV.—*A Case of Medullary Sarcoma of the Liver, with Abdominal Tumor, involving the Heart and Left Kidney.* By JOHN P. KLUGE, M.D., Physician to the Panama R. R. Company, and formerly Resident Physician at the Emigrant's Refuge Hospital, New York.

ADAM HELLER, aged twenty-seven, German, trade a blacksmith, was admitted into ward 18 of the Emigrant's Refuge Hospital, Ward's Island, New York, Nov. 26th, 1852.

*History.*—He states that he has never been seriously ill, with the exception of a periodical bloody discharge per urethram, ushered in with premonitory pains of a lancinating character in the renal regions. This sanguineous discharge or hematuria, he says, has occurred at regular intervals, varying from four to six weeks, and usually continued for three or four days, when it ceased altogether, and did not again appear until the time above mentioned had elapsed. Upon questioning him more closely, however, he informed me that two years ago he had suffered for several weeks from inflammation of the kidneys, as the physician in attendance had told him. This illness occurred prior to his coming to the United States.

Four weeks after his arrival in America he was admitted into this hospital, having, at the time, œdema of the feet, and complaining of a sharp pain in the region of the left kidney. He says that his periodical discharge has not appeared for the last three months, to which fact he ascribes his present indisposition.

Being rather anæmic and debilitated, a tonic course of treatment, consisting principally of a nutritious diet and the vegetable bitters, was adopted, and five weeks afterward he declared himself quite well, and was discharged, cured, by his own request. Now he returns again, after having been four months absent from this hospital, and after having been for five weeks previous a patient at the Quarantine Hospital on Staten Island, suffering, according to his statement, with the same complaint with which he was first admitted into the Emigrant's Hospital.

*Present Condition.*—Upon first viewing the patient after his second admission to the hospital, I anticipated a case of severe and long-continued intermittent fever. His countenance bore an anxious expression, and he was evidently suffering great pain. His complexion was sallow; pulse slow and very weak; tongue perfectly clean; appetite considerably impaired; cutaneous veins much congested, and of a dark blue color; feet œdematous.

Upon physical examination of the liver, it appeared indurated, extending to the processus eusiformis of the sternum, and half an inch below the line of the navel on the left side. Urine scanty, of a light straw color, and upon being allowed to settle deposited a whitish sediment.

*Diagnosis.*—Obscure; supposed the case to be either one of cirrhosis or carcinoma hepatis.

*Prognosis.*—Unfavorable; the engorged state of the abdominal cutaneous veins rendering it probable that some serious obstruction existed in the portal circulation.

*Treatment.*—He was again put upon a course of the bitter vegetable extracts, as on a former occasion, consisting of ext. taraxaci, nux vomica, etc. Patient, however, daily grew weaker; diarrhœa, with severe abdominal pains, set in about two weeks after his admission, and he became much reduced in flesh. Regarding the case as hopeless, and daily anticipating death to take place, I now treated only the prominent symptoms, as they presented themselves, by narcotics and anodynes, when, after a few days, a new phenomenon presented itself, in the discharge of about a pint of bloody urine; he rallied; his strength commenced to return; his urine gradually assumed a healthy character; and January 10th, 1853, he is able to sit up, and even walk about a little without assistance. He continued to improve for several weeks, when he was again attacked with diarrhœa and most of the other symptoms which presented themselves at the time of and shortly after his admission. He again recovered. This alternate recovery and relapse continued until spring, when for several months the patient steadily improved, without any unfavorable symptoms manifesting themselves, with the exception of occasional œdema of the legs and feet, occurring generally toward evening. This came and went, and was not, at the time, regarded as of much moment. He spent the whole spring and summer in the hospital, his general health being better during that time than could be expected, though it became daily more and more evident that he was laboring under a chronic disease of the liver and kidneys. Nothing of particular interest occurred until November, 1853, when the patient was again obliged to take to his bed; his feet and legs became more œdematous; the cutaneous or superficial veins of the chest and abdomen became tortuous in their course; in fact, a varicose condition of them took place, with astonishing congestion of even the most minute venous branches.

The liver, judging from the evidence afforded by the physical signs, extended as far upward as the sixth rib on the right side, compressing the lower lobe of the right lung. A severe catarrh soon set in, with troublesome diarrhœa, and the veins before referred to became engorged to a fearful degree.

The treatment adopted was merely palliative, anodynes and alkalies, which latter, in the form of Seidlitz powder, or soda bicarb., afforded him the greatest relief, so that he was continually asking for them—even when the state of his bowels was such as to render their administration unadvisable for a time. He at length became so much prostrated that, by the first of December, his pulse could scarcely be felt or counted at the wrist. He refused all nourishment, and complained of great pain in the pharynx whenever he attempted to swallow anything, even small portions of fluid matter. Now and then he could be persuaded to take a little stimulus in the form of wine-whey, or brandy-punch, or small quantities of beef-tea; and it was surprising that he should, nevertheless, continue to exist in this state for several weeks.

Death, at length, came to his relief, and terminated his sufferings on the 18th December, 1853, at 3 P.M. He died with symptoms of pulmonary œdema, which was discovered to have been the case upon making the autopsy.

*Autopsy* eighteen hours after death.—The liver was much enlarged, extending upward to the sixth rib, and compressing the inferior and middle lobes of the right lung; their color was normal, but the liver filled with circumscribed masses of medullary sarcoma, in consistence like brain substance. These masses varied in size from that of a hazelnut to that of a large walnut; and this viscus was, as it were, studded with these tumors on its surface. Closely attached to the posterior inferior portion of the liver was a large tumor, irregular in outline, hard, and covered with a serous membrane; superiorly, it was firmly attached to the base of the heart, (which was free from valvulary or other organic disease,) a little to the right of the origin of the aorta. In its posterior portion it was adherent to the spinal column, and the left kidney was entirely imbedded in it and incorporated with its substance. There was œdema of both lungs, and enlargement with softening of the spleen, which was about twice its normal size. A portion of the mesentery and pyloric extremity of the stomach adhered to the tumor. The stomach and intestines appeared to be quite healthy in other respects.

The intestines lay freely over the tumor, without any adhesions to it, and it was only detected upon raising the intestines and liver from their natural positions. A careful examination of this diseased mass demonstrated it to be, essentially, medullary sarcoma, disposed in lobes connected by areolar tissue. Its weight was six pounds, avoirdupois; circumference thirty inches, and longest diameter eight inches. The right kidney presented a normal appearance. Brain and spinal marrow not examined.

